# Transcending the brain: is there a cost to hacking the nervous system?

**DOI:** 10.1093/braincomms/fcz015

**Published:** 2019-09-16

**Authors:** Shujhat Khan, Tipu Aziz

**Affiliations:** 1 School of Medicine, Imperial College London, London SW7 2AZ, UK; 2 Department of Neurosurgery, John Radcliffe Hospital, University of Oxford, Oxford OX3 9DU, UK

**Keywords:** brain–computer interface, deep brain stimulation, transcranial direct current stimulation, ethics, law

## Abstract

Great advancements have recently been made to understand the brain and the potential that we can extract out of it. Much of this has been centred on modifying electrical activity of the nervous system for improved physical and cognitive performance in those with clinical impairment. However, there is a risk of going beyond purely physiological performance improvements and striving for human enhancement beyond traditional human limits. Simple ethical guidelines and legal doctrine must be examined to keep ahead of technological advancement in light of the impending mergence between biology and machine. By understanding the role of modern ethics, this review aims to appreciate the fine boundary between what is considered ethically justified for current neurotechnology.

## Teaser

Neurotechnology has the ability to drastically improve patient prognosis as well as providing the possibility of enhancing those who are not in clinical need for such technology. We examine ethical viewpoints discussing the potential clinical capabilities and risks that such mergence between biology and machine can pose.

## Graphical Abstract

**Graphical Abstract:**
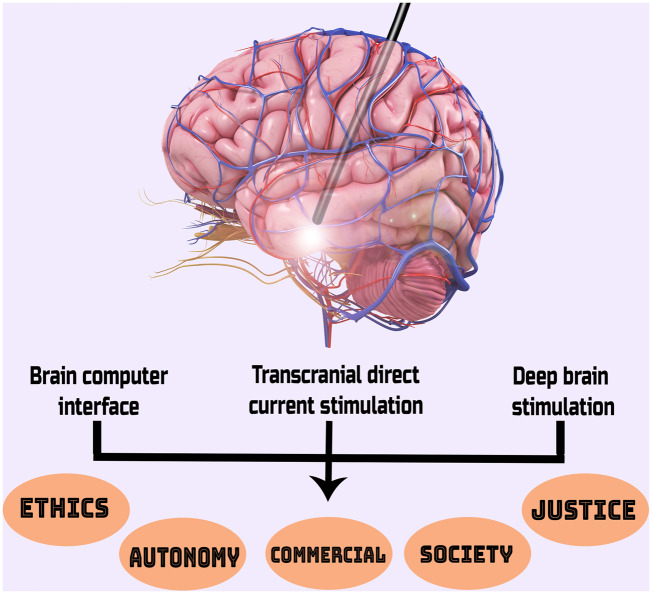

## Introduction

Fascination with the brain has existed since Napoleonic times. In one of the earliest published reports of the *Annals of surgery*, Cushing (1903), often referred to as the father of neurosurgery, reported a successful case of nerve anastomosis in the spring of 1902. Cushing’s patient, a 30-year-old male, had received a bullet wound, which entered the skull through the right-sided mastoid process and had proceeded to damage the lower part of the fallopian aqueduct, irreparably damaging the facial nerve in the process. The patient reported a number of symptoms: sensory and motor deficits to the anterior two-thirds of the tongue; right-sided lachrymation; and paralysis to the facial muscles and platysma. Cushing described the operation in detail. He administered the anaesthetic and proceeded to incise along the anterior border of the sternocleidomastoid muscle, exposing the accessory nerve before cutting through the posterior border of the parotid gland to expose the facial nerve. Upon transplantation between the facial and accessory nerves, the patient was sutured, sent home and armed with a galvanic battery for electrical stimulation to promote exercise of facial muscles. The symptoms steadily improved, facial asymmetry markedly improved and the paralysis diminished. Encouraging procedures such as this one demonstrated the willingness of pioneering surgeons to be bold, even callous, in moulding anatomy and challenging physiology. Such early fascination with controlling electrical activity of nerves was without the ethical constraints of the future but laid the foundation for modern techniques that allows manipulation of the brain without the constraints of the past.

Following Cushing’s operation, a slew of firsts quickly followed. In 1968, Wyrwicka and Sterman (1968) recorded and translated sensorimotor rhythms into sensory feedback. In 1969, Fetz demonstrated the role of operant conditioning in enabling control of single cortical neurones (Fetz and Baker, 1973). In 1973, Vidal, a UCLA professor, proposed a system whereby EEG signals could be translated into computer control signals—leading him to coin the term brain–computer interface (BCI) and giving rise to a technique to read brain signals (Vidal, 1973). As well as reading these signals, pioneers looked to control neural tissue by applying non-invasive and invasive electrical currents so as to better treat clinical conditions (Delgado *et al.*, 1952; Horgan, 2005; Utz *et al.*, 2010). Subsequently, the following years saw researchers become better at reading and influencing the electrical signals of the brain for desired effects. In this review, we discuss the ethical dilemmas associated with the use of these neurotechnologies that attempt to control neuronal electrical activity.

## Power of neurotechnology

### Uses of brain–computer interfaces

**Figure 1:**
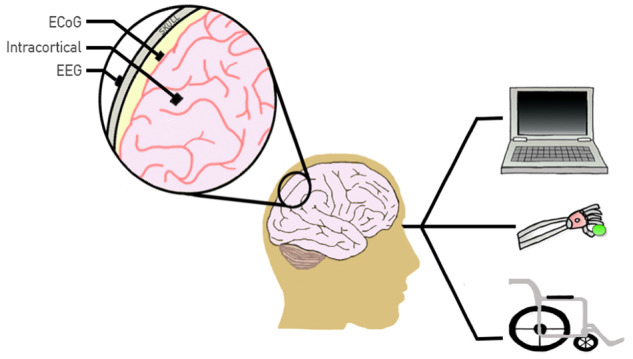
**Types of BCIs and their uses.** There are three types of BCIs. They can be non-invasive such as EEG (measured on the scalp), invasive such as electrocorticography (ECoG) (measured on the cortex) and intracortical. The electrophysiological signals can then be translated to operate devices including assistive devices to help patients with movement and communication, or for rehabilitation including helping patients recover from motor and cognitive defects. As such they can be used to support word-processing, cursor movement, control of robots and prosthetics, and motor rehabilitation (Daly and Wolpaw, 2008).

Modern BCIs have the ability to revolutionize clinical care. They can be used to treat a wide range of conditions (Fig. 1 and Table 1). Stroke, in particular, is a significant field where BCIs can have an important function. Patients are often left with motor impairments following stroke and experience balance and mobility issues. To improve prognosis from stroke disability, neural plasticity is seen as a potential rehabilitation target (Dimyan and Cohen, 2011). This might be especially beneficial for the 15–30% of stroke patients who are permanently disabled (Lloyd-Jones *et al.*, 2009) because of the ability to connect BCIs to function electrical stimulation. As such, it allows users to be able to concentrate at a greater intensity on moving affected limb, thus speeding the rehabilitation process (Daly *et al.*, 2009). This mechanism involves the BCI picking up the distinctive pattern in the motor cortex when an individual imagines a movement sequence. The information can then be translated to provide electrical stimulation of the nerves in the patient’s relevant muscle groups in order to initiate movement. In the case of traumatic spinal cord injury, BCIs are able to bypass the site of injury and have the capability of restoring restricted function. By using functional electrical stimulation, they can causes contraction of non-functioning muscles. This can have a profound impact because the incidence of traumatic spinal cord injury remains high with hundreds of thousands of new cases emerging annually (Lee *et al.*, 2014), leaving many with tetraplegia if the cervical spinal cord is affected. Not only are BCIs able to connect with functional electrical stimulation, but they can also be connected to an exoskeleton to facilitate with movement (Daly *et al.*, 2009; Garcia-Cossio *et al.*, 2015; Xin *et al.*, 2015; Frolov *et al.*, 2017; Marghi *et al.*, 2017).

**Table 1:** An overview of some of the current uses and future potentials of neurotechnologies

Technology	Current uses	Future potential
BCIs	Communication using BCIs in patients with locked-in syndrome has already been demonstrated but is at a very early stage. However, they are too slow and inaccurate for widespread use (Sellers *et al.*, 2014).	Faster and more accurate communication between users is likely to be seen in coming years. Delays longer than the order of hundredths of milliseconds can lead to less efficient BCIs and a reduced potential for therapeutic gain.
		Furthermore, once signals get accurate and quick enough, they can be used for other purposes such as entertainment including virtual reality systems amongst other applications (Kosmyna and Lécuyer, 2019).
	BCIs have already been shown to connect with exoskeletons and neuroprosthetics such as robotic hands with surprisingly impressive functionality (Hochberg *et al.*, 2012; Collinger *et al.*, 2013).	The EEG signal is currently too unreliable to be used solely in assistive devices such as wheelchairs or indeed in robotics control. Further work needs to be done to decrease the response time as well as making the signal more reliably accurate to prevent harm to the patient.
	BCIs can also be used to help with cognitive and motor rehabilitation in patients in adjunction with other devices such as functional electrical stimulation and tDCS that aids the rehabilitation process through a top-down fashion (Hong *et al.*, 2017).	Future BCIs may have the potential to control additional forces such as strength and torque. Currently BCIs are unable to pick these additional forces reliably. This can be achieved through the incorporation of kinetics in the ability of BCIs to decode brain activity. By combing this with precise residual muscle activation, patients will be able to recover quicker and have better functionality.
		Connection with the cloud can allow cellular and sub-cellular information to be stored and then used for the treatment of disease. Such data will have a high monetary and commercial value (Martins *et al.*, 2019) and the potential for sensitive data-mining and fraud.
DBS	DBS has become a standard treatment for movement disorders such as Parkinson’s disease.	A better understanding of anatomical targets within the brain will allow a better response with fewer side-effects.
	DBS can also be useful for psychiatric disorders including depression (Sartorius *et al.*, 2010), Tourette’s syndrome (Baldermann *et al.*, 2016), bipolar disorders (Gippert *et al.*, 2017), OCD (Mallet *et al.*, 2008) and anorexia nervosa (Lipsman *et al.*, 2017).	The complexities and considerable overlap that is often seen in psychiatric disorders makes this quite difficult to treat and introduces doubt when managing these patients. A more detailed understanding of the complex mechanisms by which DBS exerts its effects as well as a better knowledge of psychiatric pathophysiology will aid management. In addition, there are only a few studies looking at DBS for many of the conditions seen in psychiatry, and further studies would serve to provide a clearer picture. This is especially true of Tourette’s syndrome, where it is estimated that <300 patients have undergone DBS (Baldermann *et al.*, 2016).
	DBS is being touted for treatment in Alzheimer’s disease, driven by the ability of DBS to influence activity in key limbic circuits.	This is very much in the early stages. More research needs to be done to illustrate the potential benefits.
tDCS	tDCS is well established for being able to improve cognitive and motor effects and, therefore, serves as a useful adjunct in conditions such as stroke (Lefebvre and Liew, 2017; Shaker *et al.*, 2018).	Numerous studies have showed a large variability in results. As such, several factors need to be addressed including the precise location of the electrode placement, size of current, as well as timing of the current. This will need to be addressed further before therapeutic use (Lefebvre and Liew, 2017).
	tDCS can be helpful in psychiatric conditions such as major depressive disorder (Brunoni *et al.*, 2013).	Long-term follow-up is required to see if the effects are long-lasting. Furthermore, some patients develop worsening of their symptoms and, in the case of depression, can develop mania or hypomania following tDCS administration. Therefore, future studies need to be conducted to analyse which parameters can worsen outcomes (Brunoni *et al.*, 2013). Such parameters include the electrode position, the size of the current, duration of electrical stimulation, as well as the number of sessions they have. Furthermore, tDCS should be analysed as an adjunct to traditional medication such as anti-depressants to see if a combination of these two can have a better response.
	tDCS has also been used in the paediatric population with great success as seen in the treatment of paediatric motor disorders (Saleem *et al.*, 2019).	Long-term effects of tDCS have yet to be elucidated. Studies have already shown that tDCS has the potential to induce synaptic plasticity through epigenetic regulation (Podda *et al.*, 2016) and this can have long-term consequences for paediatric patients.

Interestingly, use of functional magnetic resonance imaging and diffuse tensor imaging demonstrated neural plasticity and recovery in stroke patients following BCI physiotherapy (Fridman *et al.*, 2004; Sharma *et al.*, 2009; Caria *et al.*, 2011), suggesting the ability to produce persistent results in function recovery as well as making the physiotherapy process more efficient.

Furthermore, BCIs can also be used for communication between two individuals through brain-to-brain interface linked to a computer-to-brain interface (Grau *et al.*, 2014). This involves reading electrical signals in the brain of the sender and appropriately stimulating the areas of the brain of the receiver to transmit information. Communication can, as a result, ensue through neural control and, therefore, allows bi-directional dialogue between two individuals who may not otherwise be able to communicate, as seen in the case of locked-in syndrome (Vansteensel *et al.*, 2016).

However, if BCIs were used in those who do not suffer from disabilities, it would allow users to surpass current human limitations and give them almost super-hero-like abilities. These might include being able to transmit information through thoughts and connect directly to prosthetics such as robotics or to computing software (Daly and Wolpaw, 2008).

### Deep brain stimulation

Deep brain stimulation (DBS) is a technique employed by functional neurosurgeons to treat a number of different disorders. It involves inserting electrodes in certain areas of the brain to produce electrical signals to affect the electrical circuitry of the brain. Perhaps most famously, it is used to treat Parkinson’s disease—a disorder, which involves loss of dopaminergic neurones in the substantia nigra pars compacta. As a result, it leads to impairment of the direct and indirect pathways of the basal ganglia leading to movement disorders, with symptoms including tremors, rigidity and akinesia (Wichmann *et al.*, 2011). Studies show that in Parkinson’s disease, there is an increased rate of neuronal firing in the globus pallidus interna, and the subthalamic nucleus but a decreased rate of firing in the globus pallidus externa (Magill *et al.*, 2001; Soares *et al.*, 2004). Whilst the exact mechanism of DBS is still unclear, it is believed to involve disruption to the abnormal basal ganglia circuitry to restore normality (Wichmann and DeLong, 2016). Alongside Parkinson’s disease, DBS is also investigated for use in depression, movement disorders, chronic pain, neuropsychiatric disorders, epilepsy, addictions and Alzheimer’s disease amongst other conditions (Table 1;Nuttin *et al.*, 1999; Volkmann *et al.*, 2012; Munte *et al.*, 2013; Delaloye and Holtzheimer, 2014; Falowski, 2015; Aldehri *et al.*, 2018; Klinger and Mittal, 2018).

It is worth pointing out that DBS is only being used on patients who are clinically in need of neurosurgical intervention for debilitating disorders such as Parkinson’s disease. However, with such promising results that DBS offers, it can become tempting to see how it can benefit healthy individuals to improve, for example, cognition and memory (Suthana *et al.*, 2012). However, this is less likely to be tried than with, for example, transcranial direct current stimulation (tDCS) because of the invasive nature and specialist neurosurgical intervention required.

### Transcranial direct current stimulation

Unlike DBS, non-invasive brain stimulation methods can also produce clinical outcomes as seen with (tDCS). This is where electrodes are positioned to target regions of the scalp and a current, with a typical magnitude of between 1 and 2 mA, passes through the skull and accesses neurones and glial cells. tDCS has an added advantage of using anodal or cathodal stimulation in order to augment neuronal excitability to produce more complex results. In a simplified model, short-term use of anodal stimulation leads to excitation whilst longer use leads to decreased excitation. On the other hand, use of cathodal stimulation provides the opposite results (Nitsche and Paulus, 2000; Batsikadze *et al.*, 2013; Monte-Silva *et al.*, 2013). Interestingly, tDCS also has the capability to excite subcortical neuronal tissue and affect memory and emotions but it is not clear whether this is through direct stimulation or indirectly from connected neurones (Im *et al.*, 2012; Bolzoni *et al.*, 2013).

The use of tDCS has been proposed for the treatment of a number of conditions (Table 1) including improving cognition, memory in Alzheimer’s and Parkinson’s diseases, neuropsychiatric disorders, chronic pain and motor impairment (Ferrucci *et al.*, 2008; Kang *et al.*, 2016; Goodwill *et al.*, 2017; Ricci *et al.*, 2018; Yesavage *et al.*, 2018).

However, its use for enhancement has already been well documented and companies have developed kits to allow customers to be able to experiment on themselves, in a bid to improve cognition or motor performance (Wexler, 2016, 2017).

## The ethical aspects of brain hacking

Neuroenhancement is when healthy individuals make pharmacological or technological changes to the brain in order to improve characteristics such as cognition or physical performance (Wexler, 2017). These individuals are not in medical need of neurotechnology unlike, for example, those suffering from cognitive impairment and as such, neuroenhancement can be seen as a way of neural doping (Davis, 2013; Marcello and Pim, 2016). This can alternatively be seen as a form of brain hacking. The definition of brain hacking is open to interpretation. On the one hand, it can be described as accessing and then manipulating neuronal information in the brains of those with BCIs (Ienca and Haselager, 2016) whilst on the other hand, it can be interpreted as attempting to increase cognitive performance (Wexler, 2017) such as through tDCS. Here, we refer to hacking as using neurotechnology to read or alter neuronal signalling to induce both therapeutic and adverse cognitive, psychological or motor effects. This, therefore, also includes the concept of brain-reading whereby individuals attempt to read user’s minds by interpreting brain signals (Mecacci and Haselager, 2019).

The vast potential of such technologies is evident. Whilst their application towards the treatment of clinical conditions is obvious, it also exposes a number of ethical dilemmas. As an example, the long-term consequences of many neurotechnologies remain untested. This includes the potential to change memory and personality—factors that are intrinsically linked to one’s identity (Ulla *et al.*, 2006; Voon *et al.*, 2008; Kraemer, 2013). In addition, neurotechnology is being investigated for use in the paediatric population. However safety parameters have not yet been established and as such can have dangerous consequences (Minhas *et al.*, 2012). With such uncertainty regarding the potential of this technology, it poses questions to research ethics committees and asks whether they are willing to accept these risks. Indeed, if neuroenhancement therapies are conducted in a clinical setting by doctors, society will likely be more willing to permit this because it can be seen as improving the well-being of the patient. Before approving this technology for medical use for both the treatment of pathological impairments as well as enhancement purposes, regulatory bodies need to look at a number of factors. These include analysis of the pathophysiology and biochemical changed induced by the technology, as well as assessing the potential long-term consequences. Furthermore, it is important to think of the effect that this can have on the society as well and how inequality in access can lead to distributive injustice—although it is worth bearing in mind that society already tolerates such injustice with accessibility to plastic surgery. In addition, once approved, clinicians themselves may have a duty to demonstrate a paternalistic approach and, much like how plastic surgery is allowed in specific cases in the national health service (NHS), neuroenhancement may be permitted following a thorough assessment of individual patients. This includes maximizing benefits and minimizing damage to the patient with the view of exercising beneficence, non-maleficence and respect for autonomy.

## Neurotechnology: the good and the bad

Neural enhancement would seem an attractive proposition to many. With the brain being the central organ to many features that individuals would like to improve, neuroenhancement would attract a lot of interest. Sports players can use it to gain an advantage over other competitors; students will be able to use it to perform better in tests; and the military can use it to gain an advantage over other countries (Fregni *et al.*, 2005; Borducchi *et al.*, 2016). Alternatively, an argument can be made for professionals such as doctors who work in high-pressure environments to have access to this technology. If these individuals are able to operate at a greater capacity for longer durations from the use of neuroenhancement, it can lead to improved outcomes for patients, and society benefits overall. In addition, medication has already been used to improve cognition (Sugden *et al.*, 2012). By using neurotechnology to achieve the same objective, it perhaps suggests that it should be viewed in the same light as medical drugs.

On the other hand, neuroenhancement can be seen as a form of technological advancement that aims to alter human nature. Here, it is important to ascertain what human nature means. Traditionally, this would involve characteristics that are common to healthy humans and distinguishes them from animals. This includes evolutionary characteristics including, but not limited to, cognitive reasoning, the ability to make moral judgements, and perhaps a superior capacity to emotional perception (Sakai *et al.*, 2003). The integration of man with machine may seek to give characteristics beyond current human parameters and towards perfectionist and hubristic notions. Current limitations that bind humans are seen by some as part of the natural order, and, according to some religious perspectives, overcoming these limitations through biotechnology can be seen as over-mastering nature and seeking that which is beyond evolutionary limitations.

## Autonomy

Autonomy in the context of neuroenhancement is particularly concerned with allowing individuals to make decisions without the risk of feelings and thoughts that one would otherwise experience through the influence of neurotechnology. During electrical stimulation from DBS, for example, patients may often feel emotions such as alienation (Kraemer, 2013), suicide (Voon *et al.*, 2008) and manic behaviour (Ulla *et al.*, 2006). Because emotions are so intrinsically linked to our decision-making, it is plausible to suggest that individuals may have impaired autonomy. As a result, it raises the question as to whether their actions are truly their own or a result of downstream consequences originating from neuro-stimulation (Karsten, 2013). Indeed, this can affect a patient’s autonomy if they are unable to willingly give adequately informed consent to their participation because the long-term consequences of this technology are not properly established. Based on this argument, we can look at the nature of autonomy and whether one may be allowed to autonomously decide to impair or even permanently abrogate their autonomy. Indeed Mill (1859) provides powerful reasoning rejecting the right of individuals to intentionally revoke their own autonomy when he says ‘the principles of freedom cannot require that the person be free not to be free’. Without truly understanding the long-term consequences of the technology and the intricacies with which the brain can be affected, it is difficult for users to make informed decision about whether or not they should utilize neurotechnology. Neurotechnology, at its core seeks to affect neurochemical processes, which can, as a side-effect, affect an individual’s role as moral agents through altered decision-making and, therefore, impaired autonomy. Moreover, in order to have true autonomy when deciding to use neurotechnology, it is important for individuals to be free from any form of pressure that can influence their decision. The philosopher Dr O’Neil (1984) notes that in order to have serious respect for autonomy, participants would at the very least be able to refuse consent to treatment. Take, for example, athletes who may be pressured into using tDCS for improving motor performance (Goodwill *et al.*, 2017) due to social and political pressures as well as internal pressure to reap reward for years of hard work. As a result, they may fortuitously impair their own autonomous decision-making.

## Commercial side-effects

### Effects on the individual

It is perhaps concerning that corporations such as Facebook are looking to delve into the field of neurotechnology. In addition, other companies such as Neuralink, Kernel and new start-ups such as Openwater are also looking to develop BCIs to allow alternative methods for faster communication in commercial applications (Takmakov, 2017). It would be naïve not to consider the ethical repercussions that could occur from this. Whilst Facebook is largely looking to increase the number of eligible users to include paralyzed individuals who would thus be able to access their services for communication through BCIs, further societal acceptance may mean that third-party access to brain technology can lead to potential collection of neural information as a means of consumer targeting. Indeed, large corporations already provide third-party access, usually through customer’s unknowing approval, that infringes on patients’ right to privacy (International Committee of Medical Journal Editors, 1995; Grundy *et al*., 2017). In addition, neural signals can be linked to an individual’s identity, which disregards patient confidentiality and is a clear privacy concern (Koike-Akino *et al.*, 2016). This is important because privacy is fundamental to allow individuals to exercise true autonomy devoid of social and political pressure. Furthermore, it is possible to get information through BCIs that can be extracted to reveal security breaches. This includes information related to personal information such as their personal details, health-related information, banking information, as well as political and societal beliefs that can be of interest to criminals, employers, insurance companies and corporations looking to better understand their target market (Bonaci *et al*., 2015; Marcello and Pim, 2016).

The use of tDCS kits that are available for home-use raises a number of concerns (Wexler, 2016). Self-improvement can give individuals an unfair advantage in tests and sports (Davis, 2013). Moreover, it can lead to individuals trying to increase the amplitude of the electrical current in the pursuit of more favourable results. Similarly, as DBS gets more evolved and broader areas of the brain are excited, it allows greater potential for hackers to alter the programming of the stimulation therapy. Users themselves can also act as hackers in an attempt to amplify results, for example, to increase activation from reward centres (Denning *et al.*, 2009). This sets a dangerous precedent whereby non-trained individuals may inadvertently risk causing unforeseen acute and chronic damage (Buhmann *et al.*, 2017).

In addition, hacking into BCIs can enforce limitations on users activities, which leads those affected unable to autonomously act. This can be particularly dangerous for those individuals who look to neuroenhancement to improve physical characteristics. Such individuals include those who use BCIs that are linked to prosthetics, for example, robotics limbs, who can then suddenly lose a crucial part of their functioning (Daly and Wolpaw, 2008; Hochberg *et al.*, 2012; Vansteensel *et al.*, 2016). In this situation, it is important to remember the psychological impact that this can have on these users and their ability to trust neurotechnology in future instances. Furthermore, it can lead to a loss of trust in neurotechnology from the general public, which can make future advancements more difficult.

### Effects on the society

There is a lot of interest in the functionality of the brain—understandably so considering the large burden of neurological diseases that the world faces (Chin and Vora, 2014). Fundamental to this approach is the human brain project (Amunts *et al.*, 2016)—a 10-year research collaboration costing approximately 500 million euros to comprehensively understand the enigmas of the brain. Likewise, the USA has committed to spend $4.5 billion on neuroscience research (Kaiser, 2014). In addition to this, the Defence Advanced Research Projects Agency, a unit within the US department of defence, is also exploring the use of neurotechnologies. There are a number of agendas that Defence Advanced Research Projects Agency works on including prosthetic limbs, BCI technology and electrical stimulation. These are largely aimed at restoring function following trauma but they also develop programmes involved with improving human training and performance of healthy individuals (Miranda *et al.*, 2015). These are likely intended to provide military advantage over other countries. However, if the technology becomes available, a case can be made arguing for societies’ rights to this technology. As such, the obvious question arises as to who should provide this service. In the UK, for example, the healthcare system is free through the NHS. If neuroenhancement is to be seen as a healthcare provision that should be provided for all, is it the duty of the government to ensure everyone has access to it? This would mean valuable resources are diverted away from more life-threatening diseases and disabilities. Alternatively, if neuroenhancement is given through private corporations or privatized healthcare systems as seen in the USA, it may mean that those who are poorer are unable to afford this, which creates a monetary divide within the society.

To continuously monitor progression of neuroenhancement technology and allow for sustained improvement, there will undoubtedly be an unprecedented level of data generated. This includes user data as well as data relating to the functionality of the technology. Once the datum is in the digital ecosystem, it can be subject to data-mining that will be useful for private corporations who can use it to improve marketing. In fact, the idea of understanding neural concepts to influence consumers has increased its profile rapidly, so much so it has its own field now—aptly termed neuro-marketing (Ariely and Berns, 2010). However, this raises a number of ethical dilemmas. In particular, there is a grey area where it may be difficult to distinguish between favourable clinical outcomes justified on scientific grounds as opposed to neuro-marketing by corporations savvy enough to exploit those who are vulnerable. This ties in with the dual-use concerns in neural hacking, that is, that the same technology can have both beneficial medical uses as well as detrimental consequences (Pustovit and Williams, 2010). For instance, improving cognition in those who are cognitively impaired is clearly beneficial but the commercialization of such technology for enhancement purposes would be ethically wrong.

## Justice

### Distributive justice

Whilst it is of grave importance to continue funding such endeavours to solve perhaps the greatest mystery of all, the human mind, one must also be cautious of the inclination to enhance as opposed to treat. Enhancements of the human body through plastic surgery or performance enhancing drugs are already readily accepted in society and commercialization of neurotechnology would be eagerly welcomed in the private sector. As a result, those who are able to pay for this neurotechnology will be able to improve characteristics such as cognition or motor performance to give them an advantage over their peers. Already, there is a debate over the use of prescription stimulants for cognitive enhancement in students. Many students believe that this gives their peers an unfair advantage and should be banned (Partridge *et al.*, 2013). As such, from a utilitarian perspective, it can be argued that this divide between societies can lead to far greater negative consequences than any potential positive outcome from this neurotechnology.

It is important to remember how social and ethical norms can often change depending on the circumstances and political climate. In today’s political landscape, with such global turmoil and emphasis on maximizing military advantages, there seems to be a large drive to utilize neurotechnology (Miranda *et al.*, 2015). We are perhaps now presented with a novel challenge in honing our understanding about the impact of neurotechnologies. Furthermore, the balance between government and private sector control over the use of medical advancements needs to be weighed up. Whilst in America, the private sector has a much greater influence than in European countries, it does seem that the financial stress that European countries are facing will likely give private companies more control in the health sector. As such, focus may, however undesirable, shift from ethical conduct to monetary income. This poses creating a large divide between socio-economic classes and a slippery slope towards a dystopian future.

### Legal justice

Concern regarding hacking medical devices has now been around for a number of years. Indeed, there is already potential for such security breaches in other electronic devices such as insulin pumps (Khera, 2017) as well as pacemakers or defibrillators (Clery, 2015). The brain, however, is a much more complex organ. As such, hacking neural devices can produce complex and unforeseen consequences. As an example, the new generation of DBS devices now rely on closed-loop circuitry whereby sensors are able to detect electrical signals and appropriately adjust the stimulation (Parastarfeizabadi and Kouzani, 2017). Whilst this makes the devices more efficient, it also exposes potential to hack the feedback circuitry and affect the intended functions of the device. In the case of a closed-circuit DBS system, it may be more difficult to discover hacking has taken place because of the difficulty to distinguish between hacking-induced effects and those produced as a side-effect of the device (Lavazza, 2018).

As neuroenhancement gains popularity, ethical concerns need to be confronted which can challenge established legal doctrine. Much of the legality surrounding neuroprosthetics is controlled by regulatory agencies such as the Food and Drug Administration or the European Medicines Agency. These bodies aim to heavily scrutinize healthcare products before approving them for patient use. However, technology used for neuroenhancement has complex moral importance for social injustice centred on unequal access to neurotechnology within society and, therefore, by treating this issue subjectively, it risks relativism in an incredibly complex issue. The introduction of new neurotechnologies will inevitably bring new legal challenges, and deciding how this technology can be used and what portion of the society can be afforded this technology must be addressed.

## Future directions

Medical advancement often follows the brutality and violence of war. Wars in Iraq and Afghanistan have led to a large number of injuries—changes that have spurred the field of neurotechnology. It is evident that the potential for BCIs is vast. However, our understanding of the intricacies of the brain is limited, exposing us to potential long-term consequences and ethical dilemmas. It is clear that the future of BCIs is looking towards a more synchronized alliance between human and machine. Freud (1962), a clinical neurologist, famously wrote ‘Man has, as it were, become a kind of prosthetic God. When he puts on all his auxiliary organs he is truly magnificent; … Future ages will bring with them new and possibly unimaginably great advances in this field of civilization and will increase man’s likeness to God still more’. With many looking to neurotechnology as the next step in mankind’s evolution, it is important to remember that great harm can come from this technology. Many questions related to its long-term use are still unanswered and the possibility to cause a monetary division within society means that it is important to be cautious before allowing humans to use this technology as a tool for enhancement.

Prognostication perhaps seems too early. Nonetheless, the possibilities are tantalizing. With this in mind, the Nuffield Council of Bioethics (2013) have already proposed guidelines discussing neurotechnology for enhancement and treatment purposes. However, with differing laws in different countries it is important to have a global consensus on the approach of neurotechnology for enhancement—that is, before we face the repercussions of hacking the nervous system.

## Search strategy and selection criteria

References for this review were identified by searches of PubMed and books between 1735 and 2018. The search terms ‘brain–computer interface’, ‘communication’, ‘legal’, ‘neuroprosthetics’, ‘neural rehabilitation’, ‘paralysis’, ‘bioelectronics’, ‘ethical’, ‘trauma’, ‘brain injury’, ‘electrical stimulation’, ‘genetic’, ‘history’, ‘neuroengineering’, ‘tdcs, ‘fes’ and ‘dbs’ were used. There will be no language restrictions.

## Abbreviations

BCI =: brain–computer interface
DBS =: deep brain stimulation
tDCS =: transcranial direct current stimulation
